# Assays to Detect West Nile Virus in Dead Birds

**DOI:** 10.3201/eid1111.050806

**Published:** 2005-11

**Authors:** Ward B. Stone, Joseph E. Therrien, Robert Benson, Laura Kramer, Elizabeth B. Kauffman, Millicent Eidson, Scott Campbell

**Affiliations:** * New York State Department of Environmental Conservation, Albany, New York, USA; † New York State Department of Health, Albany, New York, USA; ‡ Suffolk County Department of Health, Suffolk, New York, USA

**Keywords:** West Nile virus, birds, diagnosis, American Crow, real-time RT-PCR, dispatch

## Abstract

Using oral swab samples to detect West Nile virus in dead birds, we compared the Rapid Analyte Measurement Platform (RAMP) assay with VecTest and real-time reverse-transcriptase–polymerase chain reaction. The sensitivities of RAMP and VecTest for testing corvid species were 91.0% and 82.1%, respectively.

Since the discovery of West Nile virus (WNV) in New York in 1999, an integral part of monitoring has been testing dead bird tissue by using real-time and standard reverse-transcriptase–polymerase chain reaction (RT-PCR) (*1**–**3*). The detection limit for WNV by both methods is as low as 0.08 PFU (1.9 log_10_ PFU/mL), which indicates that RT-PCR is more sensitive than cell culture and more accurately indicates infection, since RNA is more stable than infectious virus in tissues (*3*). Recent studies have assessed potential time- and cost-saving alternatives such as VecTest (Medical Analysis Systems Camarillo, CA, USA) (*4**–**9*). Although studies have found that VecTest, with a detection limit in mosquitoes of 5.17 log_10_ PFU/mL (*10*), is less sensitive than RT-PCR for detecting WNV, test sensitivity was generally high when testing swab samples from corvid species (*4**,**6**,**8**,**9*) and certain noncorvid species such as House Sparrows (*Passer domesticus*) (*4*) and North American Owls (family *Strigidae*) (*7*). Disadvantages were occasional atypical results, including false-positives (*4*).

In this study we evaluated another alternative for WNV detection, the Rapid Analyte Measurement Platform (RAMP, Response Biomedical Corp, Burnaby, British Columbia, Canada). Limited studies conducted at the Centers for Disease Control and Prevention and the Canadian National Microbiology Laboratory indicated that the RAMP WNV test, with detection limits in mosquitoes as low as 3.17 log_10_ PFU/mL, was more sensitive than VecTest (*10*). Both tests incorporate immunochromatographic test strips by using labeled antibodies to detect antigen in samples. VecTest uses antibodies bound to gold sol particle labels, while the RAMP test uses antibodies bound to fluorescently labeled latex particles. Development of a visible reddish-purple line in both the test and control zones on the VecTest strip indicates a positive result. The RAMP test strip, enclosed within a cartridge, is inserted into a reader that calculates the ratio between the fluorescence emitted at the test and control zones and displays the results as RAMP units. Values above a background threshold are recorded as positive.

This study compared WNV results from the RAMP and VecTest on oral swab samples from dead birds, with RT-PCR on brain tissue as the standard. Brain swab samples were also tested as an alternate antigen source in the RAMP and VecTest.

## The Study

Birds included in this study were received from mid-May to late November 2004 and from mid-February through May 2005 from counties in New York State. Oral swab samples for the RAMP and VecTest were collected with 2 sterile, polyester fiber-tipped plastic applicators held together and moved around the oral cavity and proximal esophagus. One swab sample was twirled in 1.0 mL of VecTest buffer solution in a 5-mL plastic tube. The second swab sample was either twirled in 1.0 mL of RAMP buffer solution in a separate 5-mL plastic tube or placed in an empty 5-mL plastic tube, capped, and frozen at –20°C for later testing. RAMP tests were run the same day on fresh material or later on frozen samples. Before being tested, all frozen samples were thawed at room temperature; swabs not previously mixed in solution and swabs from thawed carcasses were then mixed in RAMP buffer solution. Samples were taken from the brains of a subset of corvid species by swabbing cerebral parenchyma and processing as for oral samples. The RAMP and VecTest were run according to manufacturers' directions in a class II biosafety cabinet at the New York State Department of Environmental Conservation's Wildlife Pathology Unit. RAMP test values >50, calculated by the RAMP reader, were recorded as positive. Differences in test performance were assessed by chi-square analysis. Data are expressed as a percentage in text and tables only when n is >10.

Brain samples for RT-PCR were taken at necropsy and frozen at –20°C. Brain tissue was analyzed at the Arbovirus Laboratory, Wadsworth Center, New York State Department of Health, as described previously (*2**,**3*). RT-PCR was repeated on 54 birds for which results from RAMP, VecTest, or both, contrasted with RT-PCR results. Retests of 6 birds yielded different results from the original tests. Three of these were initially positive and retested negative; the original values were low, which indicated infectivity was focal and undetected on a different sample, and the level was below RAMP and VecTest limits of detection. Three originally negative samples retested positive; 2 were highly positive, which indicated a technical error, and 1 kidney tissue sample was positive, although results of a retest with brain tissue were negative.

In this study, oral samples from 679 birds were tested; 193 (28.4%) were WNV-positive by RT-PCR. RAMP sensitivity was 80.8%, compared to 71.0% for VecTest (Table 1). For corvid species (n = 156), RAMP sensitivity (91.0%) was significantly greater than that of VecTest (82.1%) (p<0.025). With smaller sample sizes at the species level, sensitivity between RAMP and VecTest did not differ significantly (p>0.05) for 128 American Crows (*Corvus brachyrhynchos*) (91.4% and 84.4%, respectively) and 27 Blue Jays (*Cyanocitta cristata*) (88.9% and 70.4%, respectively) tested, nor for interspecies differences within each test. The detection thresholds of these tests, coupled with viral titers of specimens, may explain these different results.

**Table 1:** Oral swab RAMP and VecTest sensitivity for real-time RT-PCR–positive birds. New York, 2004–2005*

Species (presented in taxonomic order)	N†	No. positive (%)	
		RAMP	VecTest
Pelicaniformes			
Double Crested Cormorant (*Phalacrocorax auritus*)	1	0	0
Falconiformes			
Cooper's Hawk (*Accipiter cooperii*)	4	0	0
Red-tailed Hawk (*Buteo jamaicensis*)	2	0	0
American Kestrel (*Falco sparverius*)	1	1	1
Charadriiformes			
Ring-billed Gull (*Larus delawarensis*)	1	1	0
Strigiformes			
Great Horned Owl (*Bubo virginianus*)	3	1	0
Passeriformes			
Eastern Kingbird (*Tyrannus tyrannus*)	1	0	0
Red-eyed Vireo (*Vireo olivaceus*)	1	0	0
Blue Jay (*Cyanocitta cristata*)	27	24 (88.9)	19 (70.4)
American Crow (*Corvus brachyrhynchos*)	128	117 (91.4)	108 (84.4)
Fish Crow (*Corvus ossifragus*)	1	1	1
American Robin (*Turdus migratorius*)	7	1	1
Gray Catbird (*Dumetella carolinensis*)	1	0	0
Northern Mockingbird (*Mimus polyglottos*)	2	1	1
Cedar Waxwing (*Bombycilla cedrorum*)	1	1	1
Northern Cardinal (*Cardinalis cardinalis*)	1	0	0
Common Grackle (*Quiscalus quiscula*)	3	3	2
House Finch (*Carpodacus mexicanus*)	2	0	0
House Sparrow (*Passer domesticus*)	6	5	3
Total all species	193	156 (80.8)	137 (71.0)

*RAMP, Rapid Analyte Measurement Platform; RT-PCR, reverse transcription–polymerase chain reaction. †No. of birds real-time RT-PCR–positive.

RAMP confirmed more Common Grackles (*Quiscalus quiscula*) (3/3) and House Sparrows (5/6) as positive than did VecTest (2/3 and 3/6, respectively). With the exception of a few species, both tests performed poorly overall on small sample sizes of other noncorvid species.

To determine if RAMP results were affected by freezing the sample, samples from 13 corvids (10 positive, 3 negative) were retested by using swabs taken from frozen carcasses. Six initially were tested with fresh swabs and 7 with frozen swabs; all retests yielded results similar to initial results. The same results for fresh versus frozen samples were obtained with VecTest (*4*).

VecTest specificity with oral swabs was excellent in correctly identifying all 486 RT-PCR–negative birds, returning no false-positive results (Table 2). RAMP had high specificity for American Crows (98.5%), Blue Jays (90.9%), and noncorvid species (98.9%).

**Table 2:** Oral swab RAMP and VecTest specificity for real-time RT-PCR–negative birds, New York, 2004–2005*

Species (presented in taxonomic order)	N†	No. negative (%)	
		RAMP	VecTest
Galliformes			
Ruffed Grouse (*Bonasa umbellus*)	12	12	12
Columbiformes			
Mourning Dove (*Zenaida macroura*)	24	24	24
Rock Dove (*Columba livia*)	12	12	12
Passeriformes			
Blue Jay (*Cyanocitta cristata*)	22	20 (90.9)	22
American Crow (*Corvus brachyrhynchos*)	198	195 (98.5)	198
Fish Crow (*Corvus ossifragus*)	2	2	2
American Robin (*Turdus migratorius*)	22	20 (90.9)	22
Gray Catbird (*Dumetella carolinensis*)	14	13 (92.9)	14
European Starling (*Sturnus vulgaris*)	11	11	11
Brown-headed Cowbird (*Molothrus ater*)	10	10	10
Common Grackle (*Quiscalus quiscula*)	19	19	19
House Sparrow (*Passer domesticus*)	22	22	22
Other species‡	118	118	118
Total all species	486	478 (98.4)	486

*RAMP, Rapid Analyte Measurement Platform; RT-PCR, reverse transcription–polymerase chain reaction. †No. real-time RT-PCR–negative birds. ‡52 species, representing 14 orders.

Brain swab samples from 39 corvids were tested; 27 (69.2%) were RT-PCR–positive. Both RAMP and VecTest performed well, with sensitivities of 92.6% and 88.9%, respectively, and no false-positive results.

## Conclusions

Although RAMP was more sensitive than VecTest, both appear adequate for WNV surveillance in dead corvids. RAMP also performed well with oral swabs from Common Grackles and House Sparrows, although sample sizes were small. These findings are similar to previous results for VecTest, which also tested well with House Finches (*Carpodacus mexicanus*), Northern Cardinals (*Cardinalis cardinalis*), and American Kestrels (*Falco sparverius*) (*4*). As in the previous study, both tests did poorly in RT-PCR–positive raptors.

In the previous New York study, VecTests successfully tested brain, kidney, blood, feather pulp, and cloacal samples from corvids and House Sparrows (*4*). In the current study, RAMP and VecTest worked well with brain as the antigen source. Brain swab samples may be the preferred antigen source when the oral cavity is compromised. Further testing of alternative swab samples is warranted and may identify a superior antigen source; however, testing internal organs may pose greater risks and may not be applicable in field work and nonlaboratory-based surveillance. In addition, further testing, including immunohistochemical tests, on noncorvids should be conducted to accurately assess these tests and identify the distribution of WNV in the oral cavity and internal tissues.

In this study, VecTest produced no false-positive results. Although its specificity was high, RAMP produced 8 false-positive results (range 50.9–147.6). Four of these were near the >50 positive indicator level and may have been due to other sources of fluorescence. The remaining 4 false-positives (3 American Crows and 1 Blue Jay), with scores from 74.4 to 147.6, came from birds with oral cavities compromised by blood or fly eggs, which may have biased results.

VecTest results are easily distinguished when a true WNV-positive reaction occurs, but the reddish-purple line may appear faint or thin in other cases and may be subject to interpretation (*4*). RAMP quantitative results eliminate subjective interpretation, which helps assure replication but limits confidence in lower RAMP-positive scores.

The RAMP system requires an initial purchase of an electronic reader (≈US $3,500), and materials cost $13–$15 per test; VecTest costs $8 per test. If large numbers of specimens are tested, the cost of the RAMP reader per test is minimal. The RAMP test requires a minimum of 1.5 h to run because of the required cartridge drying time; VecTest takes 15–30 min to run after the test strip is placed in the sample solution.

In conclusion, both RAMP and VecTest are useful alternatives to RT-PCR for WNV surveillance in dead corvids and some passerine species when immediate turn-around of large numbers of specimens is valuable. Testing with RAMP is advantageous because of its increased sensitivity; however, follow-up testing with RT-PCR is recommended for low RAMP-positive results near the positive indicator level. Using both tests in a system in which initial testing is conducted with VecTest may also be useful; RAMP could be reserved for high-priority cases in which VecTest results are negative. RT-PCR should still be used to confirm initial viral activity in a new period and area and for research requiring more definitive results.
